# Mechanism Analysis of *OsbHLH34-OsERF34* Mediated Regulation of Rice Resistance to Sheath Blight

**DOI:** 10.3390/ijms26052249

**Published:** 2025-03-03

**Authors:** Hongyang Zhai, Chang Zhou, Yating Zhang, Yiming Wang, Mengyu Wang, Songhong Wei, Tianya Li

**Affiliations:** Department of Plant Pathology, Plant Protection College, Shenyang Agricultural University, Shenyang 110866, China; 2022220506@stu.syau.edu.cn (H.Z.); 13191598602@163.com (C.Z.); yatingz2022@163.com (Y.Z.); 2023220524@stu.syau.edu.cn (Y.W.); 2024220545@stu.syau.edu.cn (M.W.)

**Keywords:** *Rhizoctonia solani*, *OsbHLH34*, *OsERF34*, transcription factor

## Abstract

Transcription factors are pivotal molecules involved in transcriptional and post-transcriptional regulation in plants, playing a crucial role in combating biological stress. Here, we have characterized a regulatory factor, *OsbHLH34*, which governs the response of rice to infection by *Rhizoctonia solani* AG1-IA. The expression of *OsbHLH34* significantly impacts the susceptibility of rice to *Rhizoctonia solani* infection. Through the generation of *OsbHLH34* knockout and overexpressing rice plants, we observed that *OsbHLH34* acts as a positive regulator of rice resistance against rice sheath blight. The average lesion area of overexpression plants was 14.3%, the average lesion area of wildtype plants was 36%, and the average lesion area of mutant plants was 67.6%. Transcriptome and qRT-PCR analysis showed that OsbHLH34 regulates *OsERF34*, which is a key transcription factor for ethylene biosynthesis and resistance to sheath blight. By employing yeast one-hybrid and dual luciferase assays, we demonstrated that *OsbHLH34* directly interacts with the promoter of *OsERF34*, thereby activating its transcription. Both in vitro and in vivo experiments confirmed *OsERF34* as a direct target of *OsbHLH34*. These findings not only enhance our understanding of the molecular mechanisms underpinning rice disease resistance but also offer novel targets for the improvement of rice disease resistance through breeding strategies.

## 1. Introduction

Rice sheath blight disease (ShB), caused by the soil-borne fungus *Rhizoctonia solani*, results in substantial yield losses worldwide [1,2]. Yield reduction due to sheath blight disease ranges from 8% to 50%, depending on the disease severity, crop stage of infection, and environmental factors [3]. The intensification of rice planting density and nitrogen fertilizer application since the “Green Revolution” of the 1960s has elevated ShB from a minor to a major disease [4,5]. In China, between 2010 and 2020, the prevalence and yield losses due to ShB have progressively risen. Moreover, prolonged use of chemical fungicides has led to the development of fungicide tolerance, posing challenges in ShB management [6]. Therefore, developing ShB-resistant rice varieties is imperative as an alternative approach for effective ShB control. Resistance to sheath blight disease in rice is conferred by quantitative trait loci (QTLs), controlled by multiple genes [7]. Various QTLs associated with resistance to *Rhizoctonia solani* have been identified, mapped, and functionally characterized in different rice cultivars. The molecular mechanisms underlying rice resistance to sheath blight disease have been extensively studied, with pathogenesis-related (PR) genes playing a crucial role in plant defense [8]. Particularly, the PR5 family gene OsOSM1 has been validated to enhance rice resistance against sheath blight disease. OsACS2 is an important gene in rice, which encodes 1-aminocyclopropane-1-carboxylic acid synthase (ACC synthase) and is the rate-limiting enzyme in the ethylene biosynthesis pathway. Overexpressing the OsACS2 gene enhances ShB resistance [9], while a recent GWAS revealed that ZmFBL41 [7], an F-box protein, interacts with and degrades ZmCAD, an enzyme involved in lignin biosynthesis, thereby suppressing ShB resistance [10].

Transcription factors are crucial molecules in signal transduction and transcriptional regulation in plants, exerting a significant regulatory influence on plant resistance to biotic stress. Previous studies have highlighted the pivotal involvement of various transcription factor families, including WRKY, MYB, B3, bHLH, ERF, and NAC, in modulating rice resistance to sheath blight [11]. WRKY transcription factors represent a prominent family within the plant transcription factor repertoire. Characterized by the presence of the N-terminal heptapeptide motif WRKYGQK and the C-terminal zinc finger motif (C2H2 or C2-HC), this family is known for its pivotal involvement in regulating plant–pathogen interactions [12,13,14]. Jasmonic acid (JA) and ethylene (ET) are two important hormones in plants. They play a key role in plant growth and development, environmental adaptation, and defense response. OsWRKY80 boosts rice resistance to sheath blight by controlling OsWRKY4 to trigger JA/ET-dependent defense mechanisms [15]. When faced with pathogen pressure, WRKY45 and WRKY62 combine to form heterodimers, stimulating the transcription of defense-related genes like PR1b, PR1a, CPS2, CPS4, and PBZ1, thereby enhancing host resistance [16]. During the interaction between rice and *Rhizoctonia solani*, WRKY53 activates SWET2a to increase the susceptibility of rice [17].

bHLH has been implicated in plant disease resistance, with rice capable of synthesizing diterpenoid phytoalexins (DPs). The DPF gene (diterpenoid phytoalexin factor) in rice encodes a bHLH transcription factor and primarily regulates the biosynthesis of diterpenoid phytoalexins (DPs) [18]. Overexpressing the DPF gene in transgenic lines markedly boosted the levels of DP synthesis genes, rice lactone, and plant cassia. This suggests that the DPF gene acts as a positive regulator of DP accumulation. It can bind to the promoters of COPALYLDIPHOSPHATESYNTHASE2 (CPS2) and CYTOCHROMEP450MONOOXYGENASE99A2 (CYP99A2) via N-box elements to enhance the expression of these genes. The resulting products play crucial roles in the biosynthesis of plant casson and lactone [19]. It is proved that bHLH transcription factors are crucial in plant disease resistance and immune defense responses [20,21,22,23,24,25].

During rice–pathogen interaction, rice proteins interact to enhance immune pathways and regulate resistance to sheath blight. SWEET proteins in rice are a class of important sugar transporters that are widely involved in plant growth and development, stress responses, and the transport and distribution of sugars. Yang [26] discovered that the pathogen-secreted activator of SWEET2a (AOS2) interacts with WRKY53 and GT1 in rice, forming a transcription complex. This complex activates SWEET expression, facilitating the transport of carbohydrate compounds from host cells to the external environment, enabling pathogens to acquire nutrients. Yuan [27] demonstrated that PhyB inhibits the BZR1-NAC028-CAD8B pathway to modulate rice resistance against sheath blight. Kim [28] illustrated that the tissue-specific induction of SWEET14 by DOF11 enhanced rice resistance to sheath blight. Additionally, Sun [29] discovered that LPA1 activated PIN1a, thereby enhancing rice resistance to sheath blight.

The ERF (ethylene response factor) protein family is a significant subset of the AP2/ERF transcription factor family, extensively studied in plant biology for its crucial roles in plant growth, development, and stress responses. Characterized by the presence of 1–2 AP2/ERF domains, each about 60 amino acids long, this family is divided into five subfamilies—AP2, ERF, DREB, RAV, and Soloist—based on the number of domains and the presence of additional structural features. These members not only regulate normal plant development but also significantly contribute to responses to both biotic and abiotic stresses [30]. Under abiotic stress like salinity and drought, ERF proteins boost plant resistance by controlling the buildup of osmoprotective compounds. For example, VaERF3 in mung bean and OsERF71 in rice can stimulate proline accumulation, enhancing plant resistance to these specific stresses [31]. Under cold stress, EjERF39 in loquat is induced, leading to the upregulation of the lignin synthesis gene Ej4CL1, thereby facilitating fruit lignification and mitigating cold-induced damage [32]. Additionally, ERF proteins, such as VaERF092 in grapevines, can boost plant resistance by modulating other transcription factors. VaERF092 is upregulated in response to cold stress and indirectly enhances plant resistance to low temperatures by stimulating the expression of VaWRKY33. In the face of biotic stress, ERF proteins play a crucial role in defending against fungal pathogens by upregulating defense genes. For instance, ERF96 in Arabidopsis can enhance plant resistance to pathogens by upregulating pathogenesis-related genes like PDF and PR. Moreover, ERF proteins can impede pathogen proliferation by promoting programmed cell death. However, in certain scenarios, ERF proteins may exert a negative impact on plant resistance, exacerbating plant damage. For example, under iron-deficient conditions, AtERF72 in Arabidopsis is induced and suppresses the expression of IRT1 and HA2, crucial for iron absorption, consequently impeding the iron uptake [30]. In conclusion, the significance of the ERF protein family in plant stress responses is paramount. Their involvement in such responses is multifaceted, laying a crucial foundation for enhancing crops and mitigating stress.

With the completion of the whole-genome sequencing of rice, bioinformatics analysis has revealed that there are 167 basic helix–loop–helix (bHLH) transcription factors in rice [33]. So far, only a tiny fraction of them have been studied. Based on existing research, the bHLH family has been found to play a crucial role in the growth and development of plants. At present, most studies on bHLH transcription factors focus on plants under stress conditions such as drought, low temperature, salt, and heavy metals. However, research on disease resistance is relatively scarce. Moreover, the response of bHLH transcription factors in rice after infection by *Rhizoctonia solani* remains unclear. Therefore, uncovering the functions of this family of proteins is of great significance for understanding the gene function regulatory network in rice.

Previously, some studies have reported that the rice *OsbHLH34* transcription factor family genes play a key role in regulating the resistance of rice to bacterial blight [34]. However, the functions of the *OsbHLH34* transcription factor have not been studied in depth. These findings have motivated us to conduct an in-depth exploration of the functions of this gene.

In our prior research, we characterized the bHLH transcription factor *OsbHLH34* as a positive regulator of ShB resistance in rice. Here, we delve into a comprehensive examination of the operational framework of *OsbHLH34* and investigate the functionality of the *OsbHLH34* gene to unveil its underlying molecular mechanism. Our findings offer novel perspectives on the molecular foundation of ShB resistance and present promising targets for enhancing ShB resistance through breeding strategies.

## 2. Results

### 2.1. OsbHLH34 Positively Regulates Rice Resistance to Sheath Blight

**Figure 1:**
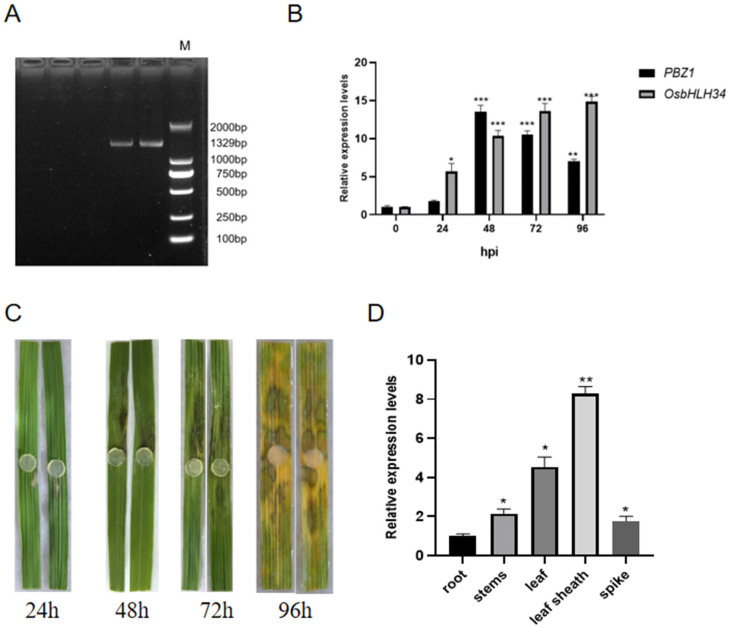
Analysis of *OsbHLH34* expression pattern (**A**) Full-length amplification of *OsbHLH34* was conducted. (**B**) The relative expression of *OsbHLH34* in rice leaves was assessed at various time points (0 h, 24 h, 48 h, 72 h, and 96 h) post-inoculation using RT-qPCR. Upregulation of *OsbHLH34* was observed in response to *Rhizoctonia solani*. (**C**) Rice detached leaves were inoculated with *Rhizoctonia solani*. (**D**) The expression of *OsbHLH34* in different tissues (leaves, leaf sheaths, roots, and stems) of rice was analyzed using RT-qPCR. Note: ns: no significance; *: *p* < 0.05; **: *p* < 0.01; ***: *p* < 0.001.

By designing primers to amplify the entire length of the gene segment of *OsbHLH34*, you are a native English-speaking American (Figure 1A).Quantitative real-time PCR (RT-qPCR) was employed in this investigation to monitor the temporal expression pattern of the *OsbHLH34* gene at various time points (0, 24, 48, 72, and 96 h) post-inoculation with AG1-IA in ZH11 leaves (Figure 1B). The findings revealed that the expression of *OsbHLH34* was upregulated in response to *Rhizoctonia solani*, with expression levels escalating over time and peaking at 96 h (Figure 1C). Analysis of *OsbHLH34* gene expression across distinct rice tissues (root, stem, new leaf, old leaf, and leaf sheath) at the mature stage demonstrated that *OsbHLH34* exhibited maximal expression in the leaf sheath, followed by the leaf blade (Figure 1D). Hence, this gene likely participates in the rice defense mechanism against sheath blight, underscoring its close association with this particular pathogen.

### 2.2. Analysis of Transcriptional Self-Activation Activity

**Figure 2:**
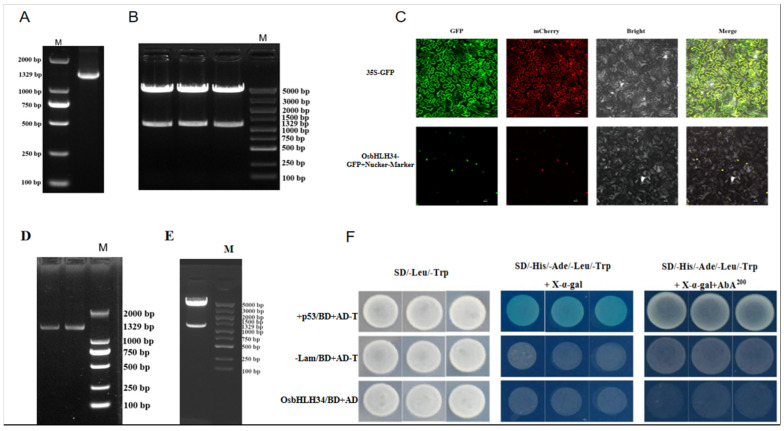
Subcellular localization and transcriptional self-activation analysis. (**A**–**C**) Subcellular localization analysis of *OsbHLH34* revealed co-localization with the nuclear marker protein H2B-mRFP1. Co-expression of *OsbHLH34*-EYFP and the nuclear marker H2B-mRFP1 in tobacco leaves resulted in co-localization. The four panels displayed from left to right show yellow fluorescence protein (YFP), red fluorescence protein (RFP), digital image channel (DIC), and a merged image of the three channels. Scale bar: 20 μm. (**D**–**F**) Growth assays on SD/−His/−Ade/−Leu/−Trp + X-α-gal medium and SD/−His/−Ade/−Leu/−Trp + X−α−gal + AbA200 medium indicated that *OsbHLH34* protein did not exhibit transcriptional self-activation in yeast cells, as it failed to grow normally and did not induce a blue coloration.

Transcriptional self-activation may affect the whole transcriptional regulatory network by changing the expression level of transcription factors. Further, the self-activation of transcription factors may lead to an increase in its concentration in the cell, thereby changing its interaction with other transcription factors and affecting the expression of other genes. To assess the transcriptional activity of *OsbHLH34* in yeast cells, the *OsbHLH34* coding sequence was fused with the GAL4 DNA-binding domain of the pGBKT7 vector. The resulting recombinant vector, pGBKT7-*OsbHLH34*, was then introduced into the yeast strain Y2HGold. Positive (pGBKT7-53 + pGBKT7-T) and negative controls (pGBKT7-Lam + pGBKT7-T) were also included. Transformants containing pGBKT7-*OsbHLH34* grew normally on synthetic dropout (SD) medium without leucine (Leu) and tryptophan (Trp), indicating successful transformation without autotoxicity. PCR verification of selected yeast colonies confirmed the transformation accuracy. However, these transformants did not grow or show blue coloration in SD medium lacking histidine (His), adenine (Ade), Leu, and Trp, supplemented with X-α-gal, or in SD medium lacking His, Ade, Leu, and Trp, supplemented with X-α-gal and 200 ng/mL aureobasidin A (AbA). These results suggest that *OsbHLH34* does not exhibit transcriptional self-activation in yeast cells (Figure 2A–D).

### 2.3. Subcellular Localization in the Nucleus

To ascertain the subcellular localization of *OsbHLH34*, a fusion protein transient expression vector was created by combining *OsbHLH34* and the GFP gene within the pGD-GFP vector. Prior online predictions indicated a probable nuclear localization of the protein. Subsequent transient expression in tobacco leaves confirmed this prediction by demonstrating the nuclear localization of the protein, as depicted in Figure 2F.

### 2.4. Construction and Expression Analysis of Mutants and Overexpression Plants

**Figure 3:**
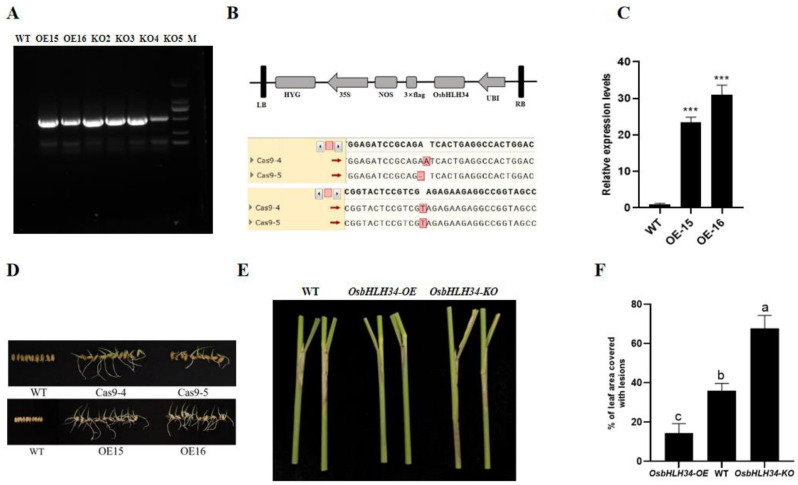
*OsbHLH34* mutant plants and overexpressed plants were obtained and verified (**A**) Amplification of hygromycin fragments in transgenic and wildtype plants was conducted. (**B**) Schematic diagrams of the overexpression vector and the *OsbHLH34* gene knockout mutant are presented. The *OsbHLH34* knockout mutants, generated using CRISPR/Cas9 genome editing, were denoted as Cas9-4 and Cas9-5. Sequencing analysis revealed the insertion of an A base and the deletion of another A base compared to the wild type. (**C**) Expression levels of *OsbHLH34* in *OsbHLH34* overexpression plants were determined using qRT-PCR. The data represent the mean ± SE of three replicates. (**D**) Screening was performed to identify hygromycin-overexpressing plants. (**E**) Leaf sheath resistance phenotypes were compared among *OsbHLH34* knockout mutants, wildtype, and *OsbHLH34* overexpression plants. (**F**) ImageJ software (v1.8.0.345) was used to measure the relative lesion area of wildtype, mutant, and overexpression plants. Note: Error bars represent means ± SE of three repeated experiments (*n* = 3). Different letters indicate significant differences (*p* < 0.05). ***: *p* < 0.001.

The PCR validation of transgenic plants overexpressing and mutated plants with hygromycin fragments indicates that both overexpression 15 and 16, and mutants 2, 3, 4, and 5 contain the hygromycin resistance fragment, suggesting successful transformation of the plants (Figure 3A). Sequencing analysis and verification of the vector structure confirm the presence of double knockout targets (Figure 3B). Expression level measurements of the overexpression plants reveal levels that are 23 and 32 times higher than those of WT plants (Figure 3C). Transgenic seeds were validated using a 100 μg/mL hygromycin solution, showing that only transgenic seeds exhibited normal growth in the solution after seven days, unlike the control group ZH11 (Figure 3D). In a live test for resistance to rice leaf blight, after 30 days of infection, the damage area of wild-type plants was significantly smaller than that of mutants and larger than that of overexpression plants, suggesting that the *OsbHLH34* gene plays a positive regulatory role in rice defense against the stripe rust fungus (Figure 3E). The area of the diseased spots of the overexpressing plant mutants and wild-type plants was determined, and the area of the diseased spots was significantly different (Figure 3F).

### 2.5. Knockout and Overexpression of OsbHLH34 Gene Did Not Affect Agronomic Traits

**Figure 4:**
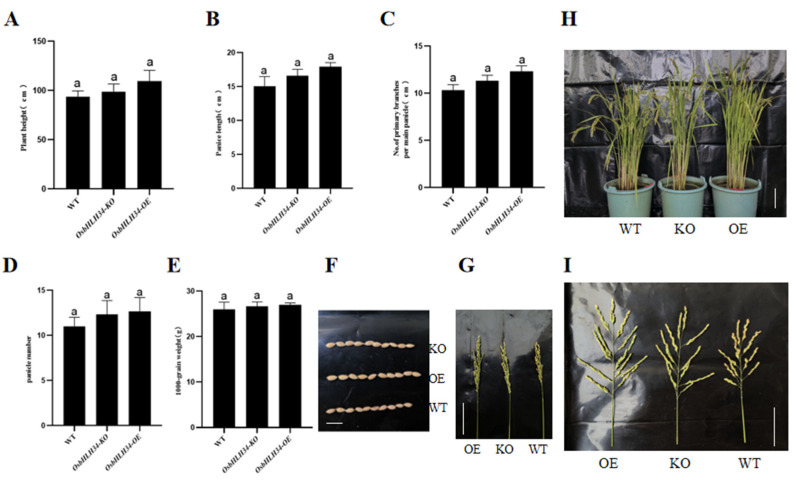
(**A**) Plant height, (**B**) panicle length, (**C**) number of primary branches per main panicle, (**D**) panicle number, and (**E**) 1000-grain weight of *OsbHLH34*-OE, *OsbHLH34*-KO, and ZH11 control. Error bars represent standard deviation. Different lowercase letters indicate significant differences (*p* < 0.05). (**F**) The scale of the photograph for 1000-grain weight.Bars =1 cm. (**G**) Number of primary branches per main panicle of *OsbHLH34*-OE, *OsbHLH34*-KO, and ZH11 control. Bars = 5 cm. (**H**) Photograph of plant height of *OsbHLH34*-OE, *OsbHLH34*-KO, and ZH11 control. Bars = 10 cm. (**I**) Number of primary branches per main panicle. Bars = 5 cm. Note: Error bars represent means ± SE of three repeated experiments (*n* = 3). Different letters indicate significant differences (*p* < 0.05).

To evaluate the utility of *OsbHLH34* genetic resources in rice breeding, we analyzed multiple agronomic and yield characteristics of the transgenic lines alongside the ZH11 control, encompassing the plant height (Figure 4A), panicle length (Figure 4B), panicle number (Figure 4C), branch number per panicle (Figure 4D), and 1000-grain weight (Figure 4E). Our findings revealed no notable distinctions between the transgenic resources and the control group (Figure 4F–I).

### 2.6. Analysis of Differentially Expressed Genes Regulated by OsbHLH34

**Figure 5:**
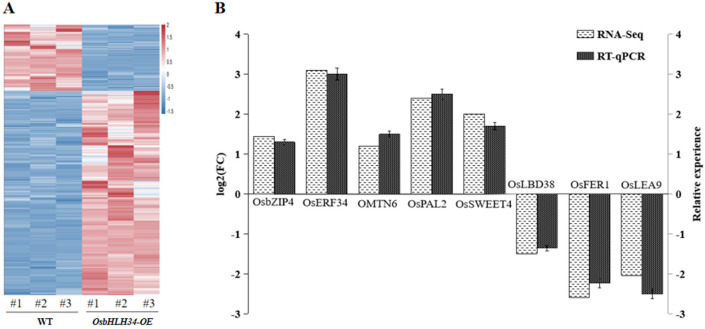
(**A**). Transcriptome heat map. (**B**). qPCR gene verification. Note: The screening criteria were |log2FC| > 1 and FDR < 0.05.

Following RNA extraction, agarose gel electrophoresis was conducted to evaluate the quality of the extracted RNA, revealing distinct bands corresponding to 28S, 18S, and 5S rRNA, indicative of successful RNA extraction and the absence of genomic DNA contamination. The samples exhibited concentrations ranging from 800 to 1000 ng/μL, as determined by microspectrophotometry, meeting the required criteria for subsequent experimental procedures. Subsequent ordinary PCR amplification using cDNA as a template demonstrated a clear and intense UBI band for the internal reference gene, indicating successful reverse transcription and equivalent amplification efficiency. The resulting cDNA was deemed suitable for further experimental analyses. Based on transcriptome data analysis, nine differentially expressed genes associated with plant disease resistance were chosen for qPCR validation. Notably, five genes (OsbZIP04, OsERF034, OMTN06, OsPAL, and OsSWEET4) that exhibited upregulation in the transcriptome were found to be positively regulated by *OsbHLH34*, showing significant differences. Conversely, four genes (OsLBD38, OsFER1, and OsLEA9) were negatively regulated by *OsbHLH34*, as illustrated in Figure 5, aligning with the transcriptome results in terms of up–down regulation trends.

### 2.7. GO and KEGG Analysis of Differentially Expressed Genes

**Figure 6:**
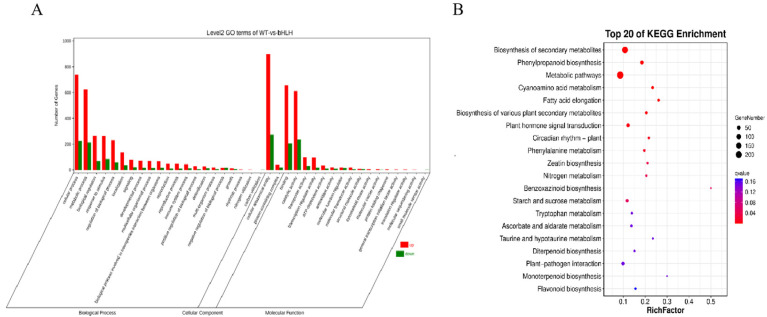
(**A**). Analysis of GO enrichment of differential genes, (**B**) Analysis of KEGG enrichment of differential genes.

A total of 2580 genes showed significant differential expression based on the established criteria in transcriptome sequencing, with 1948 genes upregulated and 632 genes downregulated. Gene Ontology (GO) analysis classified the 1948 differentially expressed genes into three functional groups: biological process, cellular component, and molecular function, as depicted in Figure 6. Among these, 21 terms were enriched in biological processes, 2 terms in cellular components, and 16 terms in molecular functions. Particularly notable enrichments were observed in binding and catalytic activities, as well as in organelles and metabolic processes. Genes with diverse functions can interact synergistically within organisms to carry out their biological functions. The Kyoto Encyclopedia of Genes and Genomes (KEGG) analysis can reveal the key signal transduction pathways and biochemical metabolic pathways associated with these differentially expressed genes. The results of the KEGG analysis suggest that a higher number of enriched genes corresponds to a more widespread distribution.

### 2.8. OsERF34 Interacts with OsbHLH34

**Figure 7:**
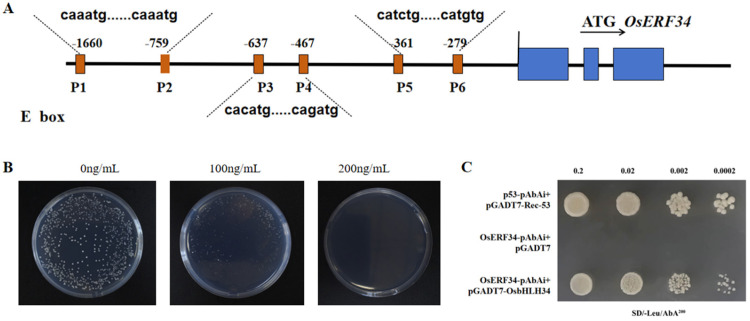
(**A**). The first 2000 bp promoter of the screened downstream target gene EFR34 was analyzed for action elements, and it was found that there were 6−10 unequal E−box elements. (**B**). AbA concentration screening of *OsERF34*−pAbAi strain (SD/−Ura medium) (**C**). Yeast one−hybrid interaction map of *OsERF34* and *OsbHLH34*.

The 2000 bp promoter region of the downstream target gene EFR34 was examined for regulatory elements, revealing the presence of 6–10 non-uniform E-box elements known to interact with bHLH transcription factors (Figure 7A). A gradient of AbA concentrations (0 ng/mL, 100 ng/mL, 200 ng/mL, 300 ng/mL, 500 ng/mL) was employed to identify the minimum inhibitory concentration against the growth of *OsERF34*-pAbAi and OsPAL1-pabai. At 200 ng/mL of AbA, no colonies were observed on SD/Ura medium for *OsERF34*-pabai, while OsPAL1 exhibited resistance and displayed self-activation. The figure illustrates the inhibitory impact of the varying AbA concentrations (0 ng/mL, 100 ng/mL, 200 ng/mL) on *OsERF34*-pabai. Consequently, an AbA concentration of 200 ng/mL was chosen for the yeast hybridization assay (Figure 7B).

The OsERF-AbAi yeast strain was cultured in YPDA medium until reaching an optical density (OD) of 0.5–0.8. Competent cells were prepared using the CoLebo kit. Subsequently, *OsbHLH34*-pGADT7 was transformed into the Y1 HGold yeast strain containing *OsERF34*-pAbAi. After incubating for 3–5 days on SD/-Leu agar plates, positive colonies were selected and grown in YPDA liquid medium to an OD600 of 0.2. The interaction strength was assessed by spotting onto SD/-Leu/AbA (200 ng/mL) plates using a 0.9% NaCl gradient dilution method. The results showed that the *OsbHLH34* and *OsERF34* promoter had strong interaction (Figure 7C).

### 2.9. Interaction Between OsbHLH34 and OsERF34 in Vivo

**Figure 8:**
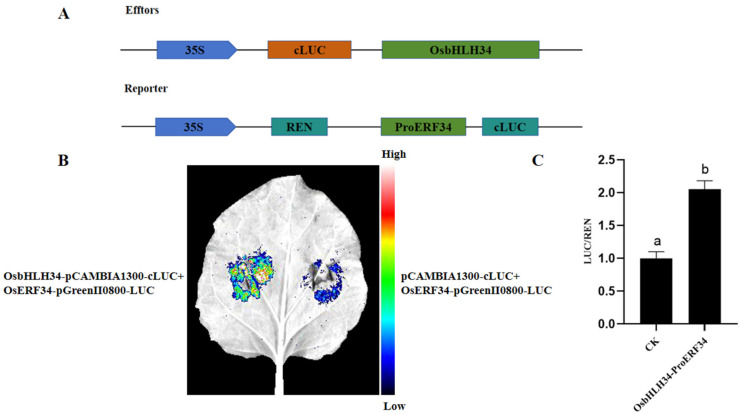
Double luciferase assay. Note: (**A**) Construction of effect carriers and reporting carriers. (**B**) Tobacco fluorescence imaging. The color band represents the fluorescence intensity. (**C**) Fluorescence detection. CK indicates the control group. Data are presented as the mean ± SE of three replicated experiments. Different letters indicate significant differences (*p* < 0.05).

Construct effect vector and report vector models (Figure 8A). To validate the findings of yeast one-hybrid screening, the dual luciferase reporter gene assay was employed to assess the interaction between the *OsERF34* promoter sequence and *OsbHLH34*. The *OsERF34* promoter fragment utilized in the single-hybrid assay was cloned into pGreenII0800-LUC to generate the reporter vector, while the coding sequence fragment of the *OsbHLH34* gene was inserted into pCAMBIA1300-cLUC to generate the effector vector, which was subsequently transformed into Agrobacterium competent cells (GV3101-psoup). Tobacco leaves were infiltrated with the reporter vector *OsERF34*-pGreenII0800-LUC and the effector vector *OsbHLH34*-pCAMBIA1300-cLUC mixed at a 1:1 ratio on the left side of the main vein; whereas on the right side, the reporter vector *OsERF34*-pGreenII0800-LUC was combined with the empty vector pCAMBIA1300-cLUC at a 1:1 ratio as a control. Following a 24 h dark treatment and 24 h light exposure, the interaction was visualized using a fluorescence imaging system. The results demonstrated that *OsbHLH34* interacted with the *OsERF34* promoter, exerting a positive regulatory effect on its transcription (Figure 8B). The dual-luciferase reporter gene assay demonstrated that compared to the negative control, the Renilla luciferase activity increased upon the interaction between *OsbHLH34* and *OsERF34* (Figure 8C).

## 3. Discussion

ShB has been a threat to rice production for many years. The ShB-resistance breeding programs have been bottlenecked due to a lack of resistant genetic resources and limited knowledge of the molecular mechanisms of rice resistance to ShB. Therefore, identifying ShB resistance regulators and dissecting their underlying mechanisms is of particular importance.

bHLH transcription factors are a widely occurring family of transcription factors in eukaryotes. They can interact with target genes through specific amino acid residues, thereby regulating the expression of related genes. It has been reported that bHLH is involved in various biological processes, including metabolism, growth, and development, stress response, hormone regulation, and secondary metabolism, playing significant roles. However, the specific mechanisms of action for most bHLH transcription factors remain largely unclear. One such transcription factor, OsbHLH148, encodes a bHLH protein. The bHLH domain of the OsbHLH148 gene shares amino acid sequence homology with bHLH transcription factors previously involved in the jasmonic acid (JA) signaling pathway, such as AtMYC2 and BPEp in Arabidopsis thaliana and RERJ1 in rice [22,23,24,25]. As a component of the JA signaling pathway, OsbHLH148 can enhance drought resistance in rice. OsbHLH148 is upregulated by methyl jasmonate and ABA, and its overexpression can increase drought resistance in rice. OsbHLH148 interacts with OsJAZ1 and is degraded by the SCFCOI1 complex-mediated 26S proteasome, thereby enhancing drought resistance in rice through the OsbHLH148-OsJAZ1-OsCOI1 signaling pathway [25]. Additionally, related studies have shown that the rice gene RERT1 (OsHLH006) can also respond to the JA signaling pathway. The RERT1 gene is induced by JA and is upregulated under non-biotic stress conditions such as drought and wounding, with its upregulation being regulated by JA [24]. These genes are involved in various biological processes, including stress and hormone responses.

ERF plays a pivotal regulatory role in plant growth and development by influencing cell differentiation and tissue formation. Additionally, it is intricately involved in the biosynthesis of various plant secondary metabolites, including lignin, flavonoids, and phytoalexins. Lignin, a crucial constituent of the plant cell wall, significantly contributes to enhancing mechanical strength and pathogen resistance. In rice, *OsERF34* acts as a positive regulator of secondary cell wall (SCW) synthesis and mechanical strength in the stem, which is essential for optimal yield. This protein stimulates the expression of the morphological determinant RMD in the uppermost internodes, thereby promoting cellulose and lignin synthesis and augmenting SCW thickness. RMD, classified as a type 2 cell-forming protein (Formin), interacts directly with cell filaments and the microtubule cytoskeleton to modulate cytoskeletal morphology and participate in rice morphogenesis [35], auxin polar transport [36], life processes such as root gravity response and soil low phosphorus adaptability [37], and phototropism of aboveground fractions [38]. Mutants deficient in RMD exhibit stunted growth, slender stems, and increased susceptibility to breakage, suggesting a role for RMD in modulating plant stem strength and influencing overall physiological processes. Among other transcription factors, SPL (SQUAMOSA PROMOTER-BINDING PROTEIN-LIKE) family transcription factors are widely involved in gene expression regulation in rice. They regulate gene expression by binding to the GTAC core motif of downstream genes. Similar binding sites may exist in the promoter region of OsERF34, which is regulated by SPL family transcription factors. WRKY family transcription factors play a key role in plant immunity and stress response. They regulate downstream gene expression by binding to W-box elements. OsERF34 may be regulated by WRKY family transcription factors through a similar mechanism, thus participating in the stress response of rice.

The function of the rice transcription factor *OsbHLH34*, a member of the bHLH transcription factor family, has not been previously documented. Through the characterization of *OsbHLH34* overexpression and knockout mutants, it was identified as a positive regulator of ShB resistance. Analysis of the *OsbHLH34* transcriptome (wild type vs. overexpression) revealed significant upregulation of the *OsERF34* gene, known to play a role in plant growth regulation. Furthermore, *OsbHLH34* activates downstream genes by binding to E-box elements [39]. Analysis revealed E-box elements in the promoter region of *OsERF34*. The binding of *OsbHLH34* to the *OsERF34* promoter was confirmed through yeast one-hybrid and transient assays, demonstrating the direct activation of *OsERF34* by *OsbHLH34*. Given *OsERF34*’s role in enhancing rice stem strength through cellulose and lignin synthesis in the secondary cell wall of the panicle node via upregulation of the cytoskeleton binding protein RMD, it is hypothesized that increased *OsbHLH34* expression in *Rhizoctonia solani*-infected rice can enhance cellulose and lignin accumulation mediated by ERF, thereby improving resistance to rice sheath blight. This establishes a *OsbHLH34*-*OsERF34*-RMD regulatory pathway elucidating the molecular mechanisms underlying *OsbHLH34*-mediated resistance to sheath blight. These findings present a novel target for sheath blight resistance breeding programs and deepen our comprehension of the molecular basis of rice’s resistance to sheath blight.

## 4. Materials and Methods

### 4.1. Plant Materials, Growth Conditions, and Fungal Inoculation

Rice plants, including overexpressed and mutant varieties of ZH11 WT and *OsbHLH34* (Os02g0726700) with ZH11 as the genetic background, were cultivated as seedlings in a growth chamber under a 16 h light (28 °C)/8 h dark (25 °C) cycle with approximately 60% relative humidity. Homozygous T1 transgenic rice seedlings were employed in this investigation [40].

*Rhizoctonia solani* AG1-IA strain was cultured on solid potato sugar agar (PDA) medium at 28 °C. The rice seedlings grown for four weeks were inoculated.

### 4.2. Protein Subcellular Localization Analysis

The *OsbHLH34*-GFP construct was generated by PCR amplification of the *OsbHLH34* ORF (Os02g0726700) without the stop codon using the following primer pairs: forward 5′-GGACGAGCTCGGTACCATGCAGCTTTTCCAAGGAGAGG-3′ (KpnI site underlined) and reverse 5′-TTGCTCACCATCTCGAGCTAGCTTTTATTGCACCGCCTC-3′ (XhoI site underlined). This resulted in the fusion of GFP in-frame at the C-terminus of *OsbHLH34*. For nuclear visualization, the *OsbHLH34* ORF (Os02g0726700) without the stop codon was subsequently subcloned into the appropriate sites of the PGD-GFP vector [41]. The coding sequence of *OsbHLH34*, lacking a stop codon, was inserted into the pGD-GFP vector using homologous recombination cloning to generate the *OsbHLH34*-EYFP fusion gene. This fusion gene was then co-expressed with P19 and H2B-mRFP1 nuclear markers in tobacco leaves. Fluorescence signals were observed 48 h post-infiltration using an Olympus FV 3000 confocal microscope (Olympus, Tokyo, Japan).

### 4.3. Quantitative RT-PCR Analysis

TRIzol™ reagent was used to inoculate rice leaves that had been grown in vitro for 30 days. Total RNA was extracted from leaves, as well as from uninoculated roots, stems, leaves, and leaf sheaths at 24, 48, 72, and 96 h post-inoculation. The extracted RNA samples, totaling 3 μg, were treated with DNase I, excluding RNase, to eliminate genomic DNA. Subsequently, cDNA was synthesized using a Tian Gen DP452 kit for reverse transcription. RT-qPCR was carried out with ChamQ SYBR^®^ qPCR Master Mix (Vazyme, Nanjing, China) on a CFX 96 real-time PCR detection system (BioRad, Hercules, CA, USA). The PCR protocol comprised 40 cycles at 95 °C for 5 s, 95 °C for 10 s, and 60 °C for 15 s. The analysis was conducted with three biological replicates. Relative quantification was performed using OsACTIN 1 as an internal control for normalization through standardized calculations [42]. All primer sequences are listed in Table S1.

### 4.4. Vector Construction and Transgenic Plant Generation

The *OsbHLH34* promoter was linked to an Amtrac high-capacity gene ORF within the pEXT06/g binary vector. The primers utilized for plasmid construction can be found in Table S1. Subsequently, the *OsbHLH34* high-capacity construct was introduced into calli of the Japonica rice cultivar Zhonghua 11 (ZH11) using the Agrobacterium-mediated transformation technique [43]. The *OsbHLH34* mutant was obtained from a rice T-DNA mutant collection (http://www.wimibio.com/, accessed on 27 February 2025) [44]. The overexpression of *OsbHLH34* was induced in the rice cultivar Zhonghua 11 (ZH11) using the modified pCAMBIA1381-Ubi vector. The *OsbHLH34* overexpression vectors were constructed at HindIII/KpnI and HindIII sites, respectively. The primers utilized for constructing the *OsbHLH34* overexpression vectors are detailed in Table S1. Comparison of hygromycin sequence PCR sequencing results confirmed the presence of the hygromycin gene sequence in the transgenic lines. Sequencing of the mutant sequence revealed two mutations in the CDS region of *OsbHLH34* CRISPR-KO: a deletion or insertion of an A base and an insertion of a T base. These mutations were in line with the anticipated editing outcomes, demonstrating the successful construction of the knockout mutant.

### 4.5. Yeast Two-Hybrid (Y2H) and Yeast One-Hybrid (Y1H) Assay

The *OsbHLH34* coding sequence (CDS) was fused to the GAL4 DNA-binding domain within the pGBKT7 vector to evaluate its transcriptional activity in yeast. The resulting recombinant vector, pGBKT7-*OsbHLH34*, was then introduced into the yeast strain Y2HGold. Negative control (pGBKT7-Lam + pGBKT7-T) and positive control (pGBKT7-53 + pGBKT7-T) constructs were also transfected into yeast cells. Subsequently, the bait plasmid was integrated into the yeast strain. Yeast cells were grown on synthetic dropout (SD) media lacking leucine (Leu) and tryptophan (Trp), as well as SD media lacking Leu, Trp, histidine (His), and adenine (Ade) (SD/-Leu,-Trp,-His,-Ade), supplemented with 50 mg/mL X-α-gal. Additionally, yeast cells were cultured on SD media lacking Leu, Trp, His, and Ade (SD/-Leu,-Trp,-His,-Ade), supplemented with 50 mg/mL X-α-gal and 200 ng/mL aureobasidin A (AbA) for selection at 28 °C for 3 days [45].

The yeast one-hybrid (Y1H) assay utilized the Matchmaker™ Gold Yeast One-Hybrid System (coolaber, Beijing, China). A 206 bp fragment of the *OsERF34* promoter region, encompassing *OsERF34* cis-acting elements, was employed as bait and integrated into the pAbAi vector (pAbAi-*OsERF34*), which was subsequently introduced into the yeast strain Y1H Gold. The coding sequence (CDS) of *OsbHLH34* was fused with the pGADT7 vector to create the *OsbHLH34*-pGADT7 construct, which was then transformed into Y1H Gold competent cells containing pAbAi-*OsERF34*. Negative controls consisted of empty vectors pGADT7 and pAbAi-*OsERF34*. Yeast cells were screened on selective medium (SD) without leucine (SD/-Leu), supplemented with 300 ng/mL AbA. The experiment was conducted in triplicate to validate the reproducibility.

### 4.6. Agronomic Traits Evaluation

In March 2024, a field trial was carried out to assess the agronomic characteristics of wildtype (WT), overexpression (OE), and CRISPR/Cas9 (Cas9) plants. The trial followed a completely randomized block design with three replications at a field nursery. Five traits were evaluated continuously during the growth stages from seedling to mature seed cane: germination rate (GerminationR), tiller rate (TillerR), number of productive tillers (ProTiller), plant height (Plant Height), and stem height (Stem Height).

### 4.7. Transcriptome Sequencing Screened Downstream Genes

In this study, we employed transcriptome sequencing (RNA-Seq) to uncover the target genes regulated by *OsbHLH34* transcription factors. This involved a thorough bioinformatics analysis, validated by experimental confirmation of the identified regulatory pathways.

### 4.8. Analysis of Promoter Action Originals

Promoter activity elements were analyzed within the initial 2000 base pairs (bp) of the target gene subsequent to transcriptome screening. The PlantCARE web tool (http://bioinformatics.psb.ugent.be/webtools/plantcare/html/, accessed on 7 October 2020) was employed for this analysis. The outcomes were visually represented to detect plausible regulatory motifs. Consistent with prior studies, it has been demonstrated that E-box elements engage in interactions with bHLH transcription factors [46].

### 4.9. Transactivation Assay

The promoter regions of *OsERF34* were inserted into the pGreenII-0800-LUC vector (Plant Pathology Laboratory, College of Plant Protection, Shenyang Agricultural University, China) to create reporter constructs. These constructs were co-transfected with the effector plasmid p35S::*OsbHLH34* in tobacco leaves. Dual-luciferase assays were performed with the Luciferase Assay Kit (Promega, Madison, WI, USA). The specific primers employed for vector construction are provided in Table S1 [47].

## 5. Conclusions

In summary, overexpression of *OsbHLH34* in rice promoted the expression of *OsERF34*, which in turn promoted the accumulation of stem sclerenchyma cells and enhanced the resistance to ShB. These results, therefore, contribute a new target for the ShB-resistance breeding programs and enhance our understanding of the molecular basis of ShB rice resistance. In summary, *OsbHLH34* was significantly upregulated after *Rhizoctonia solani* AG1-IA inoculation, suggesting that *OsbHLH34* plays an important regulatory role in the interaction between rice and *Rhizoctonia solani*. In this study, *OsbHLH34* expression pattern, mutant phenotype identification, bioinformatics, and agronomic character were analyzed. Dual-luciferase assays and yeast one-hybrid were used to analyze the regulatory function of *OsbHLH34* at the gene level. *OsbHLH34* positively regulates the rice resistance to sheath blight by binding to the *OsERF34* protein and activating the binding ability of *OsERF34* protein to lignin. Disease resistance breeding should not only consider disease prevention and control but also take into account environmental adaptability and sustainability. By cultivating disease-resistant varieties adapted to different environmental conditions, pesticide use can be reduced, and environmental pressure can be reduced. The rapid development of molecular biology technology provides an unprecedented opportunity for disease resistance breeding. In the future, by integrating multi-omics technology, gene editing, molecular marker-assisted selection, and other means, combined with big data analysis and intelligent breeding, the efficiency and accuracy of disease resistance breeding will be significantly improved, providing a strong guarantee for global food security.

## Data Availability

Data are contained within the article or Supplementary Material.

## Supplementary Materials

The following supporting information can be downloaded at https://www.mdpi.com/article/10.3390/ijms26052249/s1.
