# Correction: Zhai et al. Mechanism Analysis of *OsbHLH34-OsERF34* Mediated Regulation of Rice Resistance to Sheath Blight. *Int. J. Mol. Sci.* 2025, *26*, 2249

**DOI:** 10.3390/ijms26083545

**Published:** 2025-04-10

**Authors:** Hongyang Zhai, Chang Zhou, Yating Zhang, Yiming Wang, Mengyu Wang, Songhong Wei, Tianya Li

**Affiliations:** Department of Plant Pathology, Plant Protection College, Shenyang Agricultural University, Shenyang 110866, China; 2022220506@stu.syau.edu.cn (H.Z.); 13191598602@163.com (C.Z.); yatingz2022@163.com (Y.Z.); 2023220524@stu.syau.edu.cn (Y.W.); 2024220545@stu.syau.edu.cn (M.W.)

## Reorganization of the Corresponding Authors’ Names

Based on the order of the corresponding authors, the following corrections have been made to the original publication [1]: the authors’ names and corresponding email addresses have been corrected to Hongyang Zhai, Chang Zhou, Yating Zhang, Yiming Wang, Mengyu Wang, Songhong Wei * and Tianya Li *.

Correspondence: shw@syau.edu.cn (S.W.); litianya11@syau.edu.cn (T.L.)

The authors state that the scientific conclusions are unaffected. This correction was approved by the Academic Editor. The original publication has also been updated.
